# The effects of flooding and weather conditions on leptospirosis transmission in Thailand

**DOI:** 10.1038/s41598-020-79546-x

**Published:** 2021-01-15

**Authors:** Sudarat Chadsuthi, Karine Chalvet-Monfray, Anuwat Wiratsudakul, Charin Modchang

**Affiliations:** 1 Department of Physics, Research Center for Academic Excellence in Applied Physics, Faculty of Science, Naresuan University, Phitsanulok, 65000 Thailand; 2 INRAE, VetAgro Sup, UMR EPIA, Université Clermont Auvergne, 63122 Saint Genès Champanelle, France; 3 INRAE, VetAgro Sup, UMR EPIA, Université de Lyon, 69210 Marcy l’Etoile, France; 4 Department of Clinical Sciences and Public Health, and the Monitoring and Surveillance Center for Zoonotic Diseases in Wildlife and Exotic Animals, Faculty of Veterinary Science, Mahidol University, Nakhon Pathom, 73170 Thailand; 5 Biophysics Group, Department of Physics, Faculty of Science, Mahidol University, Bangkok, 10400 Thailand; 6 Centre of Excellence in Mathematics, CHE, 328, Si Ayutthaya Road, Bangkok, 10400 Thailand

**Keywords:** Infectious diseases, Mathematics and computing

## Abstract

The epidemic of leptospirosis in humans occurs annually in Thailand. In this study, we have developed mathematical models to investigate transmission dynamics between humans, animals, and a contaminated environment. We compared different leptospire transmission models involving flooding and weather conditions, shedding and multiplication rate in a contaminated environment. We found that the model in which the transmission rate depends on both flooding and temperature, best-fits the reported human data on leptospirosis in Thailand. Our results indicate that flooding strongly contributes to disease transmission, where a high degree of flooding leads to a higher number of infected individuals. Sensitivity analysis showed that the transmission rate of leptospires from a contaminated environment was the most important parameter for the total number of human cases. Our results suggest that public education should target people who work in contaminated environments to prevent *Leptospira* infections.

## Introduction

Leptospirosis is a worldwide zoonotic bacterial disease that is particularly endemic in tropical and subtropical countries^1,2^. The infection of humans is mainly caused by direct contact with an infected animal and also by indirect contact with urine of infected animals through cuts in the skin or mucous membranes in a contaminated environment^1,3^.

In humans, the epidemic of leptospirosis occurs annually. The highest number of cases reported in Thailand is during the rainy season from mid-May to mid-October^4^. High-risk groups include farmers and other agricultural workers, who are likely to come into contact with infected animals, and contaminated wet soil and water during their daily activities^5–7^. In addition, leptospirosis in livestock is also considered an important disease, causing reproductive failures (such as abortion, embryonic death, stillbirths, and weak offspring), decreased milk production and growth rates^8–11^. A relatively high prevalence of leptospirosis has been detected in the urine of cattle and buffalo in Thailand^11^. Contact with infected livestock increases the risk of infection^12^. These spirochete bacteria are mainly transmitted through injured or cut skin in contact with contaminated water or soil. Leptospires may survive from a few weeks to almost a year in surface water or wet soil, even during the dry season^13^.

Most of the previous leptospirosis models focused on the spreading of the disease in humans and rodents^14–16^. However, compartment models of leptospirosis, with links between the host or livestock and the environment, have also been proposed. Babylon et al. presented a simple Susceptible-Infective (SI) model to describe the spreading of leptospirosis in lambs in contact with free-living leptospires^17^. A model to study the leptospire infection dynamics in Norway rats (*Rattus norvegicus*) as the reservoir host in the environment was also presented^18^. However, a more complete model should involve human, animals, and environmental compartments for leptospirosis infection dynamics. Baca-Carrasco et al. presented an SI model to study the transmission in humans and animals with the consideration of the effect of bacteria in the environment^19^. The direct transmission between animals and humans has also been explored^20^.

Thus far, those mathematical models have not considered seasonal effects, flooding, or weather conditions. Seasonal and weather conditions have been shown to be associated with an increased leptospirosis risk^12,21–24^. In addition, the livestock species, e.g., buffalo, cattle, goats, pigs, and sheep, are the animal reservoirs and contribute to the circulation of leptospirosis in humans and the environment^25,26^. Therefore, in this work, we propose different leptospirosis transmission models that consider the impact of environmental factors such as seasonal flooding and weather conditions. The reported data on human leptospirosis in Thailand was used to fit the transmission models to identify the factors that influence the leptospirosis transmission dynamics. The proposed transmission models may help to understand the processes of leptospirosis transmission in Thailand and allow more accurate predictions of future outbreaks and better control of the disease.

## Methods

### Data

In this study, reported cases of human leptospirosis were retrieved from the national disease surveillance (report 506), Bureau of Epidemiology, Department of Disease Control, Ministry of Public Health, Thailand^27^. Most positive cases of suspected leptospirosis are based on a clinical diagnosis made by attending physicians. The clinical criteria for leptospirosis were high fever, chills, headache, with at least one of the following: abdominal pain, red eyes, muscle ache, and general malaise^28^. Other criteria include dry cough or cough with bloody sputum, and an occupational history of exposure to areas with ponds or other water or environments contaminated with animal excreta^28^. Some of the suspected cases were then examined using laboratory tests such as the Latex agglutination test (LA), Dipstick test, Lateral flow test, Microcapsule agglutination test (MCAT), Immunofluorescent antibody test (IFA), Microscopic agglutination test (MAT) or ELISA for confirmation. The suspected cases were mainly reported from public hospitals with a small fraction from private hospitals.

Data collection was performed as a part of routine clinical examination procedures of the Thai Ministry of Public Health surveillance and response. Data collection was approved by the Ethics Committee of the Ministry of Public Health of Thailand. Data containing the patient's medical records, without any patient information except location, were de-identified prior to analysis.

The remotely sensed environmental data obtained included the modified normalized difference water index (MNDWI) and the Land Surface Temperature (LST). MNDWI was extracted from the data of the Moderate Resolution Imaging Spectroradiometer (MODIS) of the Terra satellite (Surface Reflectance 8-Day L3 Global 500 m SIN Grid V005 (MOD09A1)). We used band 4 (green) and band 7 (infrared) to calculate the Modified Normalized Difference Water Index (MNDWI)^29,30^. Within the area, each pixel was classified as a flooded area if the MNDWI value was greater than or equal to zero^22,29^. Permanent water bodies were masked out using QGIS version 2.8.3^31^. The number of flooded pixels was counted to calculate the index of land flooding, which was then used to calculate the percentage of the flooded area.

The LST was extracted from the MODIS Terra product (MOD11A2) with Emissivity 8-Day L3 Global 1 km, which is composed of the daily LST product (MOD11A1) with a 1 km resolution and stored on a 1 km Sinusoidal grid as the average values of clear-sky LSTs during an 8-day period^32^.

The amount of rainfall was obtained from the real-time Tropical Rainfall Measuring Mission (TRMM) Multi-Satellite Precipitation Analysis (TMPA-RT)^33^. We derived daily precipitation and daily accumulated precipitation from the TMPA product: 3B42RT^34,35^.

The initial human population data were obtained from the WorldPop database, which presents the number of people per pixel (http://www.worldpop.org.uk). The initial livestock population of each species (buffalo, cattle, goat, pigs, and sheep) was obtained from the Information and Communication Technology Center (ICT), Department of Livestock Development of Thailand at the province level (http://ict.dld.go.th).

### Model of leptospirosis transmission

A simple SIR model of two groups is used to study the transmission dynamics of leptospirosis between humans, livestock, and the contaminated environment. Susceptible humans and livestock are denoted by $S_{h}$ and $S_{a}$, respectively. $S_{h}$ and $S_{a}$ can become infected humans ($I_{h}$) and infected livestock ($I_{a}$) through contact with infected livestock and/or the contaminated environment. The infected livestock can shed leptospires into the environment and increase the number of leptospires ($L$ compartment) in that area. The contamination level of the environment can be defined by the density of leptospires. The leptospires die at a rate $\mu_{L}$. Infected humans and animals recover at the constant rates $\gamma_{h}$ and $\gamma_{a}$, respectively. Recovered humans ($R_{h}$) and recovered livestock ($R_{a}$) lose immunity at the rates $\nu_{h}$ and $\nu_{a}$, respectively. Both population sizes are assumed to be constant. In this study, we developed a transmission model based on previous studies^19,20^. The leptospirosis transmission model is described by the following set of differential equations:

1 $$\begin{aligned} \frac{{dS_{h} \left( t \right)}}{dt} & = \mu_{h} N_{h} - \beta_{ha} \left( t \right)\frac{{S_{h} \left( t \right)I_{a} \left( t \right)}}{{N_{h} }} - \beta_{hL} \left( t \right)h\left( t \right)\frac{{S_{h} \left( t \right)}}{{N_{h} }} + \nu_{h} R_{h} \left( t \right) - \mu_{h} S_{h} \left( t \right), \\ \frac{{dI_{h} \left( t \right)}}{dt} & = \beta_{ha} \left( t \right)\frac{{S_{h} \left( t \right)I_{a} \left( t \right)}}{{N_{h} }} + \beta_{hL} \left( t \right)h\left( t \right)\frac{{S_{h} \left( t \right)}}{{N_{h} }} - \gamma_{h} I_{h} \left( t \right) - \mu_{h} I_{h} \left( t \right), \\ \frac{{dR_{h} \left( t \right)}}{dt} & = \gamma_{h} I_{h} \left( t \right) - \nu_{h} R_{h} \left( t \right) - \mu_{h} R_{h} \left( t \right), \\ \frac{{dS_{a} \left( t \right)}}{dt} & = \mu_{a} N_{a} - \beta_{aa} \left( t \right)\frac{{S_{a} \left( t \right)I_{a} \left( t \right)}}{{N_{a} \left( t \right)}} - \beta_{aL} \left( t \right)h\left( t \right)\frac{{S_{a} \left( t \right)}}{{N_{a} \left( t \right)}} + \nu_{a} R_{a} \left( t \right) - \mu_{a} S_{a} \left( t \right), \\ \frac{{dI_{a} \left( t \right)}}{dt} & = \beta_{aa} \left( t \right)\frac{{S_{a} \left( t \right)I_{a} \left( t \right)}}{{N_{a} \left( t \right)}} + \beta_{aL} \left( t \right)h\left( t \right)\frac{{S_{a} \left( t \right)}}{{N_{a} \left( t \right)}} - \gamma_{a} I_{a} \left( t \right) - \mu_{a} I_{a} \left( t \right), \\ \frac{{dR_{a} \left( t \right)}}{dt} & = \gamma_{a} I_{a} \left( t \right) - \nu_{a} R_{a} \left( t \right) - \mu_{a} R_{a} \left( t \right), \\ \frac{dL\left( t \right)}{{dt}} & = \omega \left( t \right)I_{a} \left( t \right) + m\left( t \right)g\left( t \right)L\left( t \right) - \mu_{L} L\left( t \right), \\ \end{aligned}$$

where $N = S + I + R$ for livestock and human compartments.

**Figure 1:**
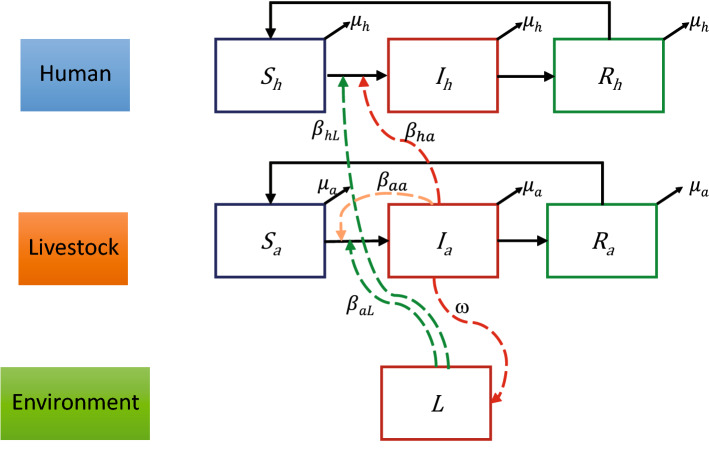
Dynamics of leptospirosis spread between humans, livestock, and the contaminated environment. The dashed green arrows show the transmission route from the contaminated environment to susceptible livestock ($S_{a}$) and human ($S_{h}$) with a transmission rate of the contaminated environment to human ($\beta_{hL}$) and to livestock ($\beta_{aL}$). Infected livestock ($I_{a}$) transmit leptospires to humans with a transmission rate of infected livestock to human ($\beta_{ha}$) and this turns a susceptible human into an infected human ($I_{h}$). The infected livestock shed leptospires to the environment with a certain shedding rate ($\omega$), shown by the red dashed line. Infected livestock ($I_{a}$) can also transmit leptospires to other livestock with a transmission rate ($\beta_{aa}$) (orange dashed line). Infected humans and animals that recover from the infections become recovered human ($R_{h}$) and recovered livestock ($R_{a}$).

In our model, we assumed that, as a zoonosis disease, the human–human transmission does not exist^8^; thus, infections in humans always occurred from animal sources or the contaminated environment. Leptospires shedding from humans into the environment is neglected in our study as the likelihood is very low. The function $g\left( t \right) = \frac{\chi - L\left( t \right)}{\chi }$ in Eq. (1) represents the logistic growth multiplier, which allows the growth to depend on the current number of leptospires and limits excessive growth, where $\chi$ is the maximum carrying capacity, or saturating population size. A saturation term, $h\left( t \right) = \frac{L\left( t \right)}{{L\left( t \right) + \kappa }}$, is added to limit the effect of transmission due to a large number of leptospires^14,17^, where $\kappa$ is the density of leptospires in the environment at which the transmission rate is $0.5\beta_{L} \left( t \right)$. A diagram of the model and its relationship between the compartments is provided in Fig. 1. The set of parameters is shown in Table 1.

**Table 1:** Descriptions and values of all parameters used in the model.

Description	Symbol	Values
Birth and death rate of humans	$1/\mu^{h}$	70 years (estimated)
Duration of infection for humans	$1/\gamma^{h}$	14 days (estimated from^3^)
Duration of loss of immunity for humans	$1/\nu^{h}$	720 days (estimated from^3^)
Transmission rate from infected livestock to human	$\beta_{ha}$	Fitted
Birth and death rate of livestock	$1/\mu^{a}$	3 years (estimated)
Duration of infection for livestock	$1/\gamma^{a}$	200 days (estimated from^36^)
Duration of loss of immunity for livestock	$1/\nu^{a}$	540 days (estimated)
Transmission rate from infected livestock to livestock	$\beta_{aa}$	Fittaed
Duration of contamination for the environment	$\mu_{L}$	0.02381 day^−1^ (estimated from^17^)
Density of leptospires at which the transmission rate from the environment is 0.5 $\beta_{L} \left( t \right)$	$\kappa$	10^2^ km^−2^ (estimated from^17^)
Maximum carrying capacity	$\chi$	1 × 10^5^ (estimated)
Density of the free living leptospires in a province at $t = 0$	$L_{i} \left( 0 \right)$	10^–3^ km^−2^ (estimated from^17^)
Density of leptospires shed per infected livestock	$\omega$	Fitted
Transmission rate from the contaminated environment to human and livestock	$\beta_{hL}$ and $\beta_{aL}$	Fitted
Multiplication rate of the leptospires in the environment	$m$	Fitted

Some of the parameters in the set of Eq. (1) may be affected by flooding and weather conditions. In this work, we look at how these conditions can affect transmission from the contaminated environment, leptospire shedding rate, and the multiplication rate.

The most important parameters for the transmission of leptospires from the contaminated environment to susceptible humans and susceptible livestock are $\beta_{hL}$ and $\beta_{aL}$. We hypothesize that the environment can influence the transmission of leptospirosis, as the virulence of leptospires depends on temperature^37^. Thus, we modified the transmission rates as a linear function of the percentage of the flooded area ($f\left( t \right)$), total monthly rainfall ($\rho \left( t \right)$), and average monthly temperature (${\rm T}\left( t \right)$). We hence test our hypothesis by examining four different scenarios in order to understand how the transmission rate depends on three environmental variables. The transmission rates of all modelled scenarios are linearly proportional to the environmental variable and are as follows:

1. Flooding (M1-F): The transmission rates are given by:
  $$\begin{aligned} \beta _{{hL}} \left( t \right) & =\, h_{1} \left( {1 + h_{2} f\left( {t - \tau _{1} } \right)} \right) \\ \beta _{{aL}} \left( t \right) & =\, a_{1} \left( {1 + a_{2} f\left( {t - \tau _{1} } \right)} \right) \\ \end{aligned}$$
2. Rainfall (M1-R): The transmission rates are given by:
  $$\begin{aligned} \beta_{hL} \left( t \right) & =\, h_{1} \left( {1 + h_{2} \rho \left( {t - \tau_{1} } \right)} \right) \\ \beta_{aL} \left( t \right) & =\, a_{1} \left( {1 + a_{2} \rho \left( {t - \tau_{1} } \right)} \right) \\ \end{aligned}$$
3. Flooding and temperature (M1-FT): The transmission rates are given by:
  $$\begin{aligned} \beta_{hL} \left( t \right) & = h_{1} \left( {1 + h_{2} f\left( {t - \tau_{1} } \right) + h_{3} {\rm T}\left( {t - \tau_{2} } \right)} \right) \\ \beta_{aL} \left( t \right) & = a_{1} \left( {1 + a_{2} f\left( {t - \tau_{1} } \right) + a_{3} {\rm T}\left( {t - \tau_{2} } \right)} \right) \\ \end{aligned}$$
4. Rainfall and temperature (M1-RT): The transmission rates are given by:
  $$\begin{aligned} \beta_{hL} \left( t \right) & = h_{1} \left( {1 + h_{2} \rho \left( {t - \tau_{1} } \right) + h_{3} {\rm T}\left( {t - \tau_{2} } \right)} \right) \\ \beta_{aL} \left( t \right) & = a_{1} \left( {1 + a_{2} \rho \left( {t - \tau_{1} } \right) + a_{3} {\rm T}\left( {t - \tau_{2} } \right)} \right) \\ \end{aligned}$$

where $h_{i}$ and $a_{i}$ are constant values of each function for each transmission rate, and $\tau_{1}$ and $\tau_{2}$ are time lags, varying from 0 to 12 weeks, which are associated with the infection of humans. Based on previous studies, we considered the effect of a time lag ($\tau$) on the environmental data in this study. *Leptospira* can survive in the autoclaved water of rice fields and pond water in Thailand for up to 12 weeks^38^. From a systematic review^39^, the survival of *Leptospira* in water was also up to 12 weeks and in soil up to 9 weeks. Rainfall has been observed to be associated with leptospirosis, often with a time lag of 1–3 months^40,41^. We, therefore, set the maximum time lag of flooding and rainfall to be 12 weeks because of the biological survivability of the bacteria in the transmission model. We set the lag period to be the same for the effects of temperature, rainfall, and flooding in this model^23^.

In the second set of models (M2-F and M2-R), leptospire shedding rates ($\omega$) were allowed to be affected by rainfall. Infected livestock shed leptospires into the environment, which will then be a source of exposure for susceptible humans and livestock. The shedding rate can be described as a logistic curve to limit its effect at high concentrations.

$$\omega \left( t \right) = \omega_{0} \left( {\frac{{\rho \left( {t - \tau_{1} } \right)}}{{\delta + \rho \left( {t - \tau_{1} } \right)}}} \right)\;{\text{and}}\;\omega \left( t \right) = \omega_{0} \left( {\frac{{f\left( {t - \tau_{1} } \right)}}{{\delta + f\left( {t - \tau_{1} } \right)}}} \right)$$

where $\delta$ is an inferred threshold parameter corresponding to the rate of half of the maximum shedding rate due to rainfall or the effect of flooding.

In the last set of models, the multiplication rate of the leptospires in the environment ($m$) depends on three environmental variables-the percentage of flooding area ($f\left( t \right)$), total monthly rainfall ($\rho \left( t \right)$), and average monthly temperature (${\rm T}\left( t \right)$). The multiplication rate is given by:

1. Flooding (M3-F): $$m\left( t \right) = x_{1} \left( {1 + x_{2} f\left( {t - \tau_{1} } \right)} \right)$$
2. Rainfall (M3-R): $$m\left( t \right) = x_{1} \left( {1 + x_{2} \rho \left( {t - \tau_{1} } \right)} \right)$$
3. Flooding and temperature (M3-FT): $m\left( t \right) = x_{1} \left( {1 + x_{2} f\left( {t - \tau_{1} } \right) + x_{3} {\rm T}\left( {t - \tau_{2} } \right)} \right)$
4. Rainfall and temperature (M3-RT): $m\left( t \right) = x_{1} \left( {1 + x_{2} \rho \left( {t - \tau_{1} } \right) + x_{3} {\rm T}\left( {t - \tau_{2} } \right)} \right)$

where $x_{1}$, $x_{2}$ and $x_{3}$ are constant values (fitted parameters).

Ten models (M1-F, M1-R, M1-FT, M1-RT, M2-F, M2-R, M3-F, M3-R, M3-FT, and M3-RT) were considered individually and compared to the null hypothesis, where all parameters are held at constant values. The effect of flooding was compared to the effect of rainfall with and without a temperature effect. A stochastic simulation approach was employed using a tau-leaping algorithm with a fixed time step^42^. Using the parameters from the best model, 1000 simulations were generated.

### Parameter estimation and sensitivity analysis

In this study, we analysed all reported cases from 2010 to 2016 from two adjacent provinces with the highest number of cases in Thailand for which reliable data were available. These provinces, Si Sa Ket and Surin, have similar climates and geographical characteristics.

To estimate the parameters of our model, we assumed that the epidemic was initiated by free-living leptospires in that area by setting the initial number of free-living leptospires to a low concentration (Table 1). To find the best-fitted model and measure the performance of the predictions, the data was divided into two sets, i.e., the reported human cases from 2010 to 2015 that fit the model and the data from 2016 which was used for prediction. The biweekly human cases from the simulation results were linked to the corresponding actual reported human cases from 2010 to 2015. The best fit was obtained by maximizing a normal log-likelihood estimation, which produced simulation results that were most similar to the reported data. We used the nlminb function in R version 4.0.2, which is a quasi-Newton method with constrained bounds, to find the optimal set of parameters^43^. The model that shows the minimum negative log-likelihood was selected as the best model.

To perform a sensitivity analysis of the modelled parameters, we used the Partial Rank Correlation Coefficients (PRCC) technique^44,45^. Then, we used Latin hypercube sampling (l h), which is a statistical Monte Carlo sampling technique, to sample the parameters using the lhs package in R^46^. 1000 parameter sets were sampled with each parameter sampled from a uniform distribution. The PRCC was ranked according to the cumulative new cases using the sensitivity package in R with bootstrapping 1000 times to obtain 95% confidence intervals^47^. Based on our assumption of linearity, positive PRCC values imply positive correlations to the response function, while negative values imply negative correlations.

### Estimation of the time-dependent reproduction number ($R_{td}$)

The basic reproduction number ($R_{0}$) is generally defined as the average number of secondary infected individuals caused by an infected individual in a population that is completely susceptible. Due to the complexity of the model and the time-dependent variables, there is no precise way to calculate $R_{0}$ as it is a complex function of many different variables. An alternative method, proposed by Wallinga et al*.*^48^, computes the time-dependent reproduction number from the observed cases using a likelihood-based method, calculated by averaging the overall transmission networks that make it fit an epidemic curve^49^. In this work, we calculated the time-dependent reproduction number ($R_{td}$) using the “R0” package in R^49^. The number of biweekly cases obtained from the simulations of the best model was used to estimate $R_{td}$. The serial interval between successive infections of the reported epidemic was identified and used to estimate the generation time distribution with the mean and standard deviation (sd) of combined data using the “R0” package. Then, the $R_{td}$ was estimated along with the 95% confidence intervals.

## Results

### Model outcomes

**Figure 2:**
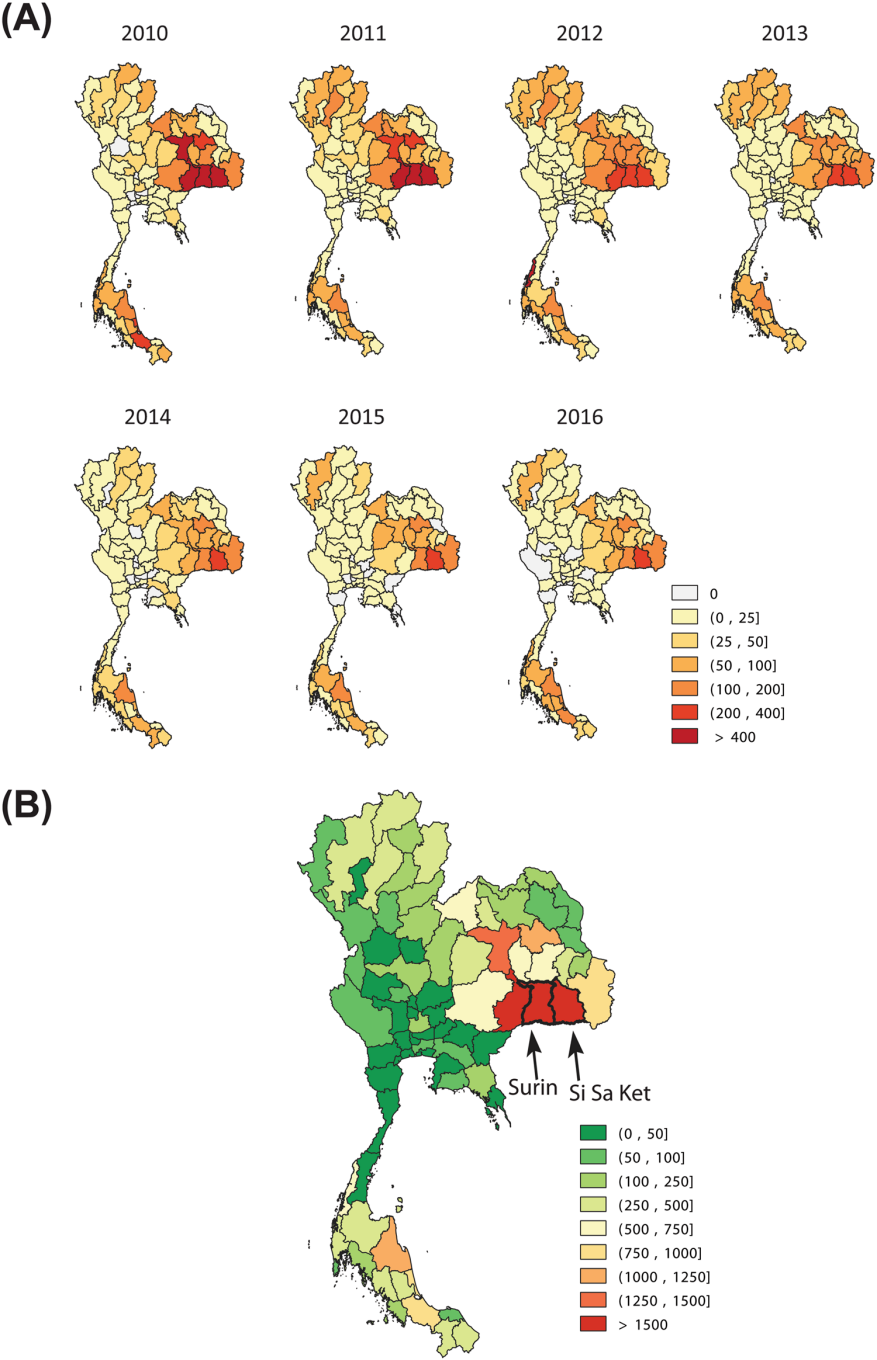
The map of reported cases in Thailand. The annual reported cases during 2010–2016 (**A**). The total reported cases during 2010–2016 (**B**).

Based on the reports of annual leptospirosis cases in Thailand from 2010 to 2016, it was found that the disease continues to spread throughout the country (Fig. 2A). High numbers of annual cases were mostly observed in the north-eastern region. In this work, we focused on two provinces that show the highest numbers of cumulative cases, i.e., Si Sa Ket and Surin (Fig. 2B). We found that the time series of biweekly reported cases in these two provinces show a similar trend (Fig. S1). The percentage of flooding and the amount of rainfall was found to increase around the same time of the year. In addition, the temperature was found to be negatively correlated with incident cases. Since it was found that Si Sa Ket and Surin have similar climate and disease transmission characteristics (Fig. S1), for simplicity, the reported data of these two provinces were combined into a single data set for further analysis.

**Figure 3:**
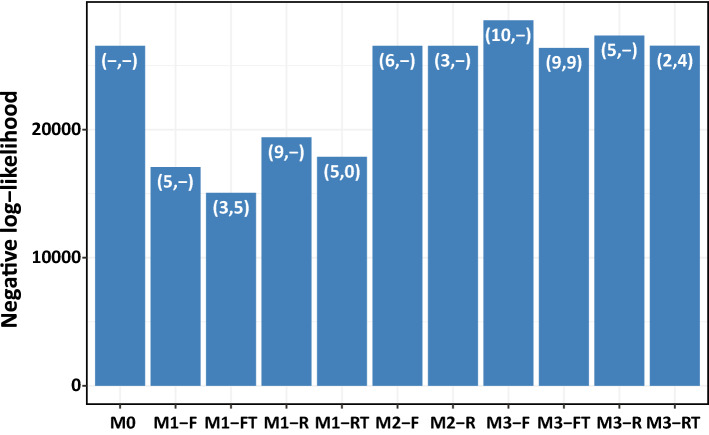
Bar chart of negative log-likelihood values for the ten models compared to a null model (M0). The parenthesis on each bar shows the time lag in weeks for flooding or rainfall and temperature (t_1_, t_2_).

We fitted eleven models (our ten models plus the null model) to the combined reported cases of Si Sa Ket and Surin from 2010 to 2015 with time lags between 0 and 12 weeks (Fig. 3). We found that the M1 models generally show better fitting performance, which indicated that the transmission rate might be linearly dependent on environmental variables, and it can highly impact the infection dynamics in humans. When comparing the models that incorporate only either a flooding factor or a rainfall factor (M1-F and M1-R), we found that the model that includes the flooding factor performs better. Moreover, incorporating a temperature factor into the models (M1-FT and M1-RT) also improved the model performance. Overall, the model with transmission rates dependent on both flooding and temperature (M1-FT) had the lowest negative log-likelihood. Thus, we selected the M1-FT model as the best-fit model for further analysis. The log-likelihood values of the M1-FT model with varied time lags of flooding showed the high likelihood at a time lag of around one month (Fig. S2). The effect of a time lag on the temperature factor was found to be different from the time lag associated with flooding.

**Figure 4:**
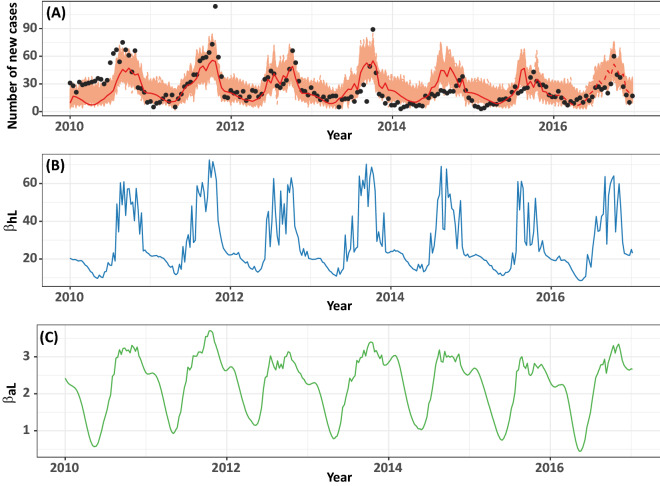
(**A**) The average number of cases obtained from the stochastic modelling of the M1-FT model (red line) compared to the reported cases of leptospirosis (black dots) for 2010–2015. The orange shaded area displays 1000 curves from the stochastic simulations. The red dashed line represents the predicted cases for 2016. The time-dependent transmission rate from the contaminated environment to susceptible human and susceptible livestock ($\beta_{hL}$ and $\beta_{aL}$) are shown in (**B**,**C**), respectively.

The stochastic modelling results of the M1-FT model, using the parameters shown in Table S1, are shown in Fig. 4A. We found that the modelling results agree with the reported data. The model prediction also provided a reasonable fit with the reported cases for 2016. The estimated time-dependent transmission rates from the contaminated environment to humans ($\beta_{hL}$) and livestock $(\beta_{aL} )$ are shown in Fig. 4B,C, respectively. We found that both of the transmission rates dramatically decline during the dry season, leading to a decline in the number of reported cases. In addition, $\beta_{hL}$ is always higher than $\beta_{aL}$ for all time, with an average value of 28.852 and 2.260, respectively. This finding indicated that the main route of human leptospirosis transmission in Thailand might be the transmission from the contaminated environment rather than from contact with infected animals.

### Time-dependent reproduction number ($R_{td}$)

**Figure 5:**
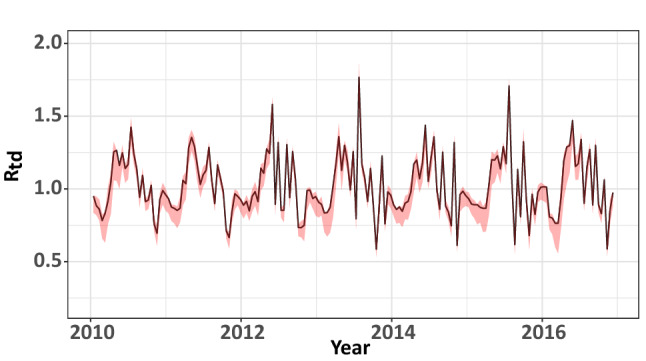
The estimated time-dependent reproduction number (solid line) with the 95% confidence interval (shaded red region).

As shown in Fig. 5, we found that the estimated time-dependent reproduction number ($R_{td}$) oscillates around 1.0, which indicates that leptospirosis is an endemic disease in Si Sa Ket and Surin provinces. The mean (sd) of $R_{td}$ is estimated at 1.021 (0.206). Normally, leptospirosis has a basic reproduction number close to zero due to its minimal transmissibility among the human population. However, this estimation could depict how leptospirosis transmits from animal sources and contaminated environments to humans.

### Sensitivity analysis

**Figure 6:**
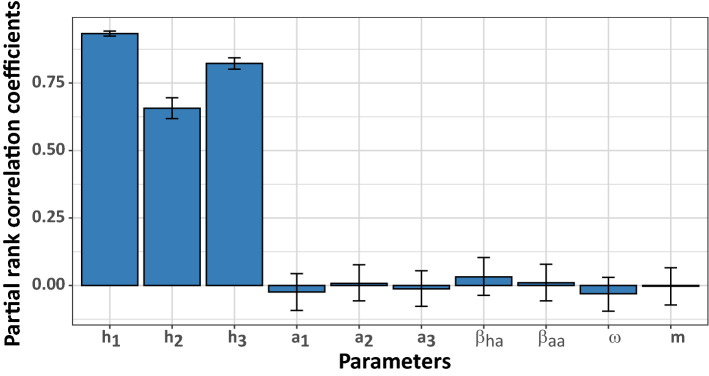
The partial rank correlation coefficients (PRCCs) of the parameters summarised in Table S1. The $h_{i}$ and $a_{i}$ are constant values of the transmission rates $\beta_{hL}$ and $\beta_{aL}$, respectively. The error bars show the 95% confidence intervals.

**Figure 7:**
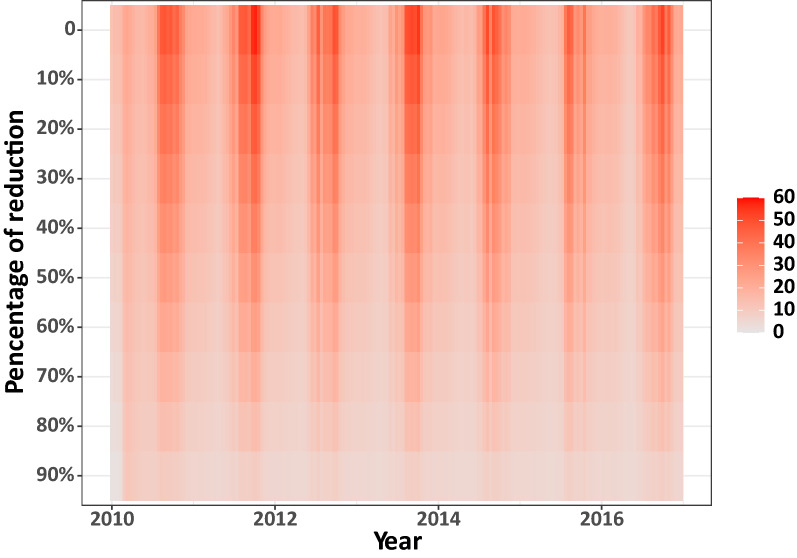
The heat map showing the number of human cases when the transmission rate from the contaminated environment to human ($\beta_{hL}$) in the M1-FT model is reduced.

Figure 6 shows the partial rank correlation coefficient (PRCC) values with 95% CI for the parameters summarised in Table S1. Here, the parameter with the absolute PRCC value greater than 0.3 was considered an important parameter. We found that $h_{1}$, $h_{2} ,$ and $h_{3}$, which are the constant values for $\beta_{hL}$, are the most important parameters. As no vaccine or specific medicines are available for leptospirosis, the most important strategy to control the disease is to reduce the leptospirosis transmission rate. Our models can also be employed to investigate how reducing the transmission rate of leptospirosis from the contaminated environment to humans can affect the leptospirosis cases in humans (Fig. 7). We found that a 90% reduction of $\beta_{hL}$, for example, could reduce the total number of human cases by ~ 90%.

## Discussion

In this work, the dynamic models incorporating the environmental data were used to investigate the transmission of leptospirosis in the north-eastern region of Thailand. This work presents the first attempt to incorporate environmental data into the mathematical modelling of leptospirosis transmission, which better describes the seasonal epidemic in north-eastern region of Thailand where there is a higher prevalence during the rainy season. Our finding suggests that transmission from a contaminated environment, as opposed to direct contact with an infected animal, is the most important driver of the leptospires outbreaks occurring in the two provinces studied. In addition, we found that the amount of flooded area in a region, which can be obtained from available satellite data, is the most important factor for leptospirosis transmission to humans. Therefore, the inclusion of an environmental leptospire compartment, which refers to the number of pathogenic bacteria in the contaminated water, might be necessary for modelling human leptospirosis infection.

Previous studies pointed out that leptospires survive and persist in water and soil for up to several weeks^39^. Environmental survival of pathogens can be an important parameter in epidemiology. During heavy rain, when flooding increases, leptospires can more easily contaminate the environment and pose a greater risk of infection through wounds on the skin. Working or living in flooded areas has been identified as a significant factor for increasing the contraction of leptospirosis^50^. In analysing our model, after fitting to human data from 2010 to 2015, the amount of flooded area was shown to be the most important parameter to improve the model compared to the rainfall. Our results are consistent with a previous study that observed animals in Thailand from 2011 to 2013^22^. This indicates that flooding is a factor that influences the epidemiology of leptospirosis in both humans and animals. Flooding was also observed to be an important risk factor in other countries such as Argentina^51^, Brazil^52^ and Malaysia^53^. In our study, including the effect of temperature improved the transmission model by only a modest amount. As a tropical country, a few degrees variance in the average temperature throughout the year is observed in Thailand. The temperature may affect leptospire virulence^37^, and the transmission rate. The temperature effect observed in our study is in line with the previous studies^23,54,55^.

In this study, the time-dependent reproduction number ($R_{td}$) was estimated for leptospirosis in humans. Normally, the basic reproduction number ($R_{0}$) of human-to-human transmission cannot be estimated due to minimal transmission between humans. However, in our study, we focused on how the transmission occurred in humans in terms of $R_{td}$. Our model’s estimation highlights that leptospirosis occurs in Thailand mainly during the mid-year period for provinces in the north-eastern region.

From the PRCC analysis, the total number of cases is mostly affected by the transmission rate of leptospires to humans. We, therefore, suggest avoiding flooded areas to reduce the transmission rate during an outbreak^56^. Moreover, protective equipment, such as boots and gloves, are recommended when in contact with flooded areas.

Although there are similar studies relating to flooded areas and weather conditions, those studies have been done in other countries which may have different climate conditions compared to Thailand. In addition, there are only a small number of studies using remotely sensed data^22,23,29,57,58^ to analyse the percentage of flooded areas. However, unlike in our study, most of the previous studies employed statistical models instead of mechanistic models. In this work, we constructed a mechanistic model to explain the relationship between meteorological sequences and the occurrence of cases. To the best of our knowledge, our model is the first compartmental model for leptospirosis transmission that considers the transmission between humans, livestock, and the contaminated environment, together with the impact of environmental factors.

Note that our proposed models were based on several assumptions, one of which is that the environmental parameters linearly affect the rates in the models. We did not consider other functions, such as a Gaussian function, due to the increased complexity this would introduce. Besides, the analysis could only be performed using the combined data of the two neighbouring provinces with the highest number of cases. The areas with medium or low numbers of cases could not be studied because the numbers of cases are too small, so that the analysis cannot provide a significant result. Our models assumed that the entire population is homogeneously mixed. Besides, we did not consider the effect of unusual weather events in our study. However, a study found that the 2011–2012 flooding in Thailand had little influence on leptospirosis transmission in those years^59^. Other animals, such as rodents, were not included due to the limitation of data on the rodent population. Other factors, such as human mobility, personal hygiene, and protective equipment, were also not accounted for in this study. The fitting process was done by only fitting to the reported human cases. Finally, the data of human cases in Thailand might not be consistently reported throughout the study time period. There might be unreported cases from the private health care centres, and asymptomatic cases might not be reported.

In summary, our study suggested that the significant environmental factor that is associated with leptospirosis transmission is flooding. A reduction in contact with a contaminated environment may help to improve disease control. Our analysis may be applied to other leptospirosis epidemic areas, where flooding data is available. Further studies should be carried out to assess the role of livestock and other relevant factors on the transmission of leptospires. Climate change or extreme weather events should also be modelled to predict the severity of future leptospirosis outbreaks. Based on our results, public health authorities may advise people who work close to or in contaminated environments to avoid contracting pathogenic leptospires in the environment and protect themselves by wearing boots to reduce the chances of leptospire contamination.

## Supplementary Information

Supplementary Information.

## Data Availability

The leptospirosis dataset used in the current study are available from Bureau of Epidemiology, Department of Disease Control, Ministry of Public Health, Thailand upon reasonable request. The datasets generated and/or analysed during the current study are available from the corresponding author upon reasonable request.
