# Supplementary material for: The effects of flooding and weather conditions on leptospirosis transmission in Thailand
Source: Sci Rep. 2021 Jan 15;11:1486. doi: 10.1038/s41598-020-79546-x (PMC7810882; doi:10.1038/s41598-020-79546-x)
Supplement: Supplementary file 1 — Supplementary Information. [file 41598_2020_79546_MOESM1_ESM.docx]

**Supplementary Information**

**The effects of flooding and weather conditions on leptospirosis transmission in Thailand**

Sudarat Chadsuthi^1,*^, Karine Chalvet-Monfray^2,3^, Anuwat Wiratsudakul^4^, Charin Modchang^5,6^

^1^Department of Physics, Research Center for Academic Excellence in Applied Physics, Faculty of Science, Naresuan University, Phitsanulok 65000, Thailand

^2^Université Clermont Auvergne, INRAE, VetAgro Sup, UMR EPIA, F-63122 Saint Genès Champanelle, France

^3^Université de Lyon, INRAE, VetAgro Sup, UMR EPIA, F-69210 Marcy l’Etoile, France

^4^Department of Clinical Sciences and Public Health, and the Monitoring and Surveillance Center for Zoonotic Diseases in Wildlife and Exotic Animals, Faculty of Veterinary Science, Mahidol University, Nakhon Pathom 73170, Thailand

^5^Biophysics Group, Department of Physics, Faculty of Science, Mahidol University, Bangkok 10400, Thailand

^6^Centre of Excellence in Mathematics, CHE, 328, Si Ayutthaya Road, Bangkok 10400, Thailand


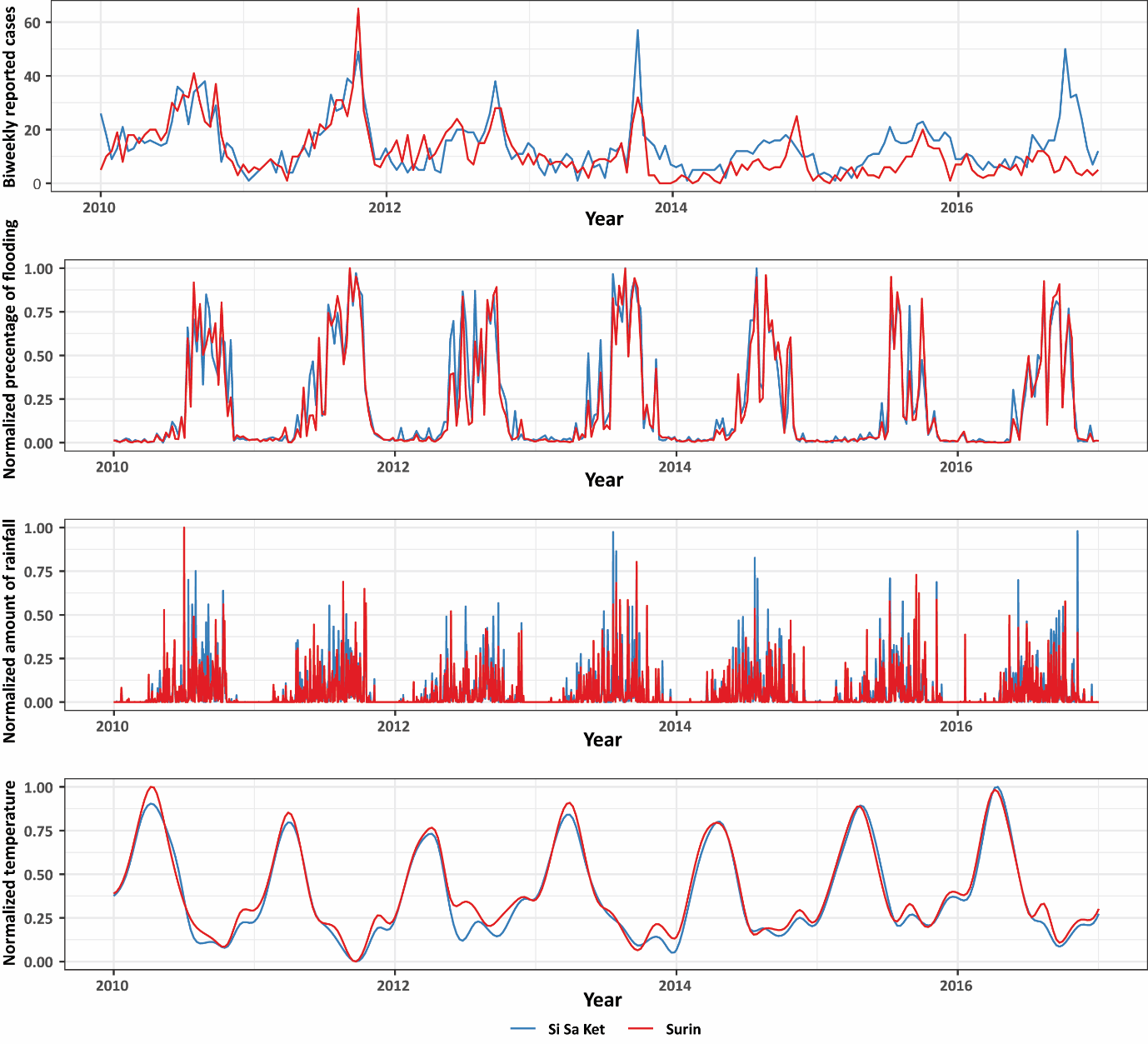


**Fig. S1.** Data collection for Si Sa Ket, and Surin provinces.


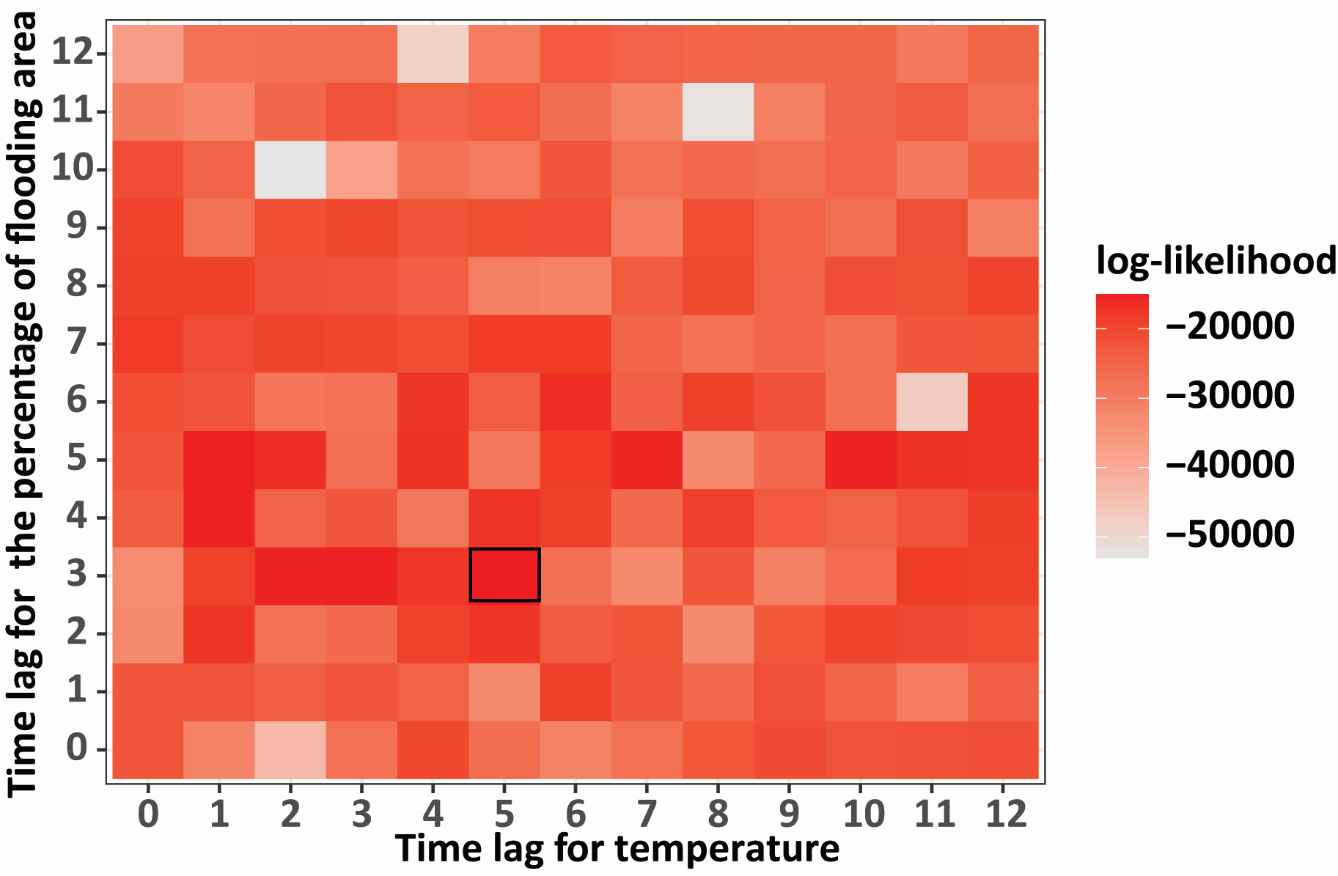


**Fig. S2.** The log‐likelihood of M1-FT model with different time lags (in weeks) using the combined data of Si Sa Ket, and Surin provinces.

**Table S1:** A summary of the estimated parameters for the M1-FT model using the combined data from 2010-2015.

| Symbol | Estimated value (95% CI) |
| --- | --- |
| $\log(\beta_{ha})$ | -5.999 (-6.050 - -5.953) |
| $\log(\beta_{aa})$ | -0.588 (-0.673 - -0.516) |
| $\log(h_{1})$ | 1.496 (1.484 - 1.508) |
| $\log(h_{2})$ | 0.189 (0.172 - 0.206) |
| $\log(h_{3})$ | -0.802 (-0.838 - -0.767)* |
| $\log(a_{1})$ | 0.636 (0.598 - 0.671) |
| $\log(a_{2})$ | -0.957 (-1.034 - -0.893) |
| $\log(a_{3})$ | -0.99 (-1.068 - -0.912)* |
| $\log(\omega)$ | -6.000 (0* - -4.293) |
| $\log(m)$ | 0.811 (0.785 - 0.836) |

*Negative number are provided on a normal scale.
